# From Suspected Brain Tumor to Cerebral Abscess: A Case Report

**DOI:** 10.7759/cureus.115571

**Published:** 2026-08-31

**Authors:** Kenza Jamai, Elmehdi Hamidi, Yassine Ait M'barek, Lamia Benantar, Khalid Aniba

**Affiliations:** 1 Neurological Surgery, Ibn Tofail Hospital, Mohammed VIth University Hospital, Marrakech, MAR

**Keywords:** brain abscess, brain tumors (primary or brain metastasis), case report, differential diagnoses, intra-axial brain tumor

## Abstract

Cerebral abscesses can mimic brain tumors or metastases on imaging, posing a significant diagnostic challenge, especially in patients without obvious infectious sources. We report the case of a 46-year-old woman with a history of spinal cord lesion biopsy, who presented with a three-week history of headaches and vomiting, progressing to dysarthria, right-sided hemiplegia, and consciousness impairment over 24 hours. On admission, she was unconscious (Glasgow Coma Scale (GCS) 7/15), with left anisocoria, requiring intubation and mechanical ventilation. Brain CT revealed intra-axial lesions suggestive of cerebral metastases. Emergency surgery was performed on the largest left frontoparietal lesion. Intraoperatively, the lesion contained purulent material, which was sterile on microbiological culture. No preoperative antibiotics had been administered. Histopathology confirmed the presence of purulent material, consistent with a cerebral abscess. Postoperatively, the patient received triple antibiotic therapy, corticosteroids, and intensive supportive care. Despite maximal treatment, there was no neurological improvement, and the patient subsequently died. This case highlights the difficulty in differentiating cerebral abscesses from metastatic brain tumors in the absence of a clear infectious source. Early consideration of abscess in the differential diagnosis and prompt surgical intervention remain critical, although outcomes may be poor in rapidly progressing cases.

## Introduction

A brain abscess is a focal suppurative infection of the brain parenchyma, characterized by a collection of pus surrounded by a vascularized capsule. Although uncommon, brain abscesses are potentially life-threatening and remain a diagnostic challenge in neurosurgical practice. Their clinical presentation is frequently nonspecific and may include headache, seizures, focal neurological deficits, altered consciousness, or signs of increased intracranial pressure [1].

Radiologically, a brain abscess may mimic a neoplastic lesion, particularly when it appears as a ring-enhancing intracranial mass [2], thereby complicating the differential diagnosis.

Distinguishing between a brain tumor and an abscess is critical, as it directly influences therapeutic management and patient prognosis [3]. We present the case of a patient initially managed for a suspected brain tumor, which was ultimately diagnosed as a cerebral abscess following intraoperative and histopathological findings. This case highlights the importance of considering infectious etiologies when evaluating intracranial mass lesions in neurosurgical practice.

## Case presentation

A 46-year-old woman, with a prior biopsy of a spinal cord lesion involving the thoracic segment (details not documented), presented with an incomplete syndrome of intracranial hypertension, characterized by headaches and vomiting for three weeks. Her condition progressed over the past week, with the onset of dysarthria and right-sided hemiplegia. The course of the disease was marked by the appearance of consciousness disorders over the past 24 hours, in a non-febrile context with marked general deterioration.

Neurological examination

The patient was unconscious upon admission, with a Glasgow Coma Scale (GCS) score of 7/15. She was promptly intubated, mechanically ventilated, and sedated. Clinical examination revealed a left anisocoria. Her vital signs were stable, with a blood pressure of 135/95 mmHg, a heart rate of 90 bpm, and an oxygen saturation of 95%. A brain CT scan demonstrated intra-axial space-occupying processes primarily suggesting cerebral secondary localizations (metastases). The contrast-enhanced axial view revealed two distinct lesions with different radiologic features (Figure 1). The sagittal reconstruction confirmed the same findings, illustrating their respective morphologic characteristics (Figure 2). In addition, another contrast-enhanced brain CT section revealed a hyperdense, poorly defined lesion with irregular margins in the right cerebellar hemisphere, associated with mild perilesional edema (Figure 3).

**Figure 1:**
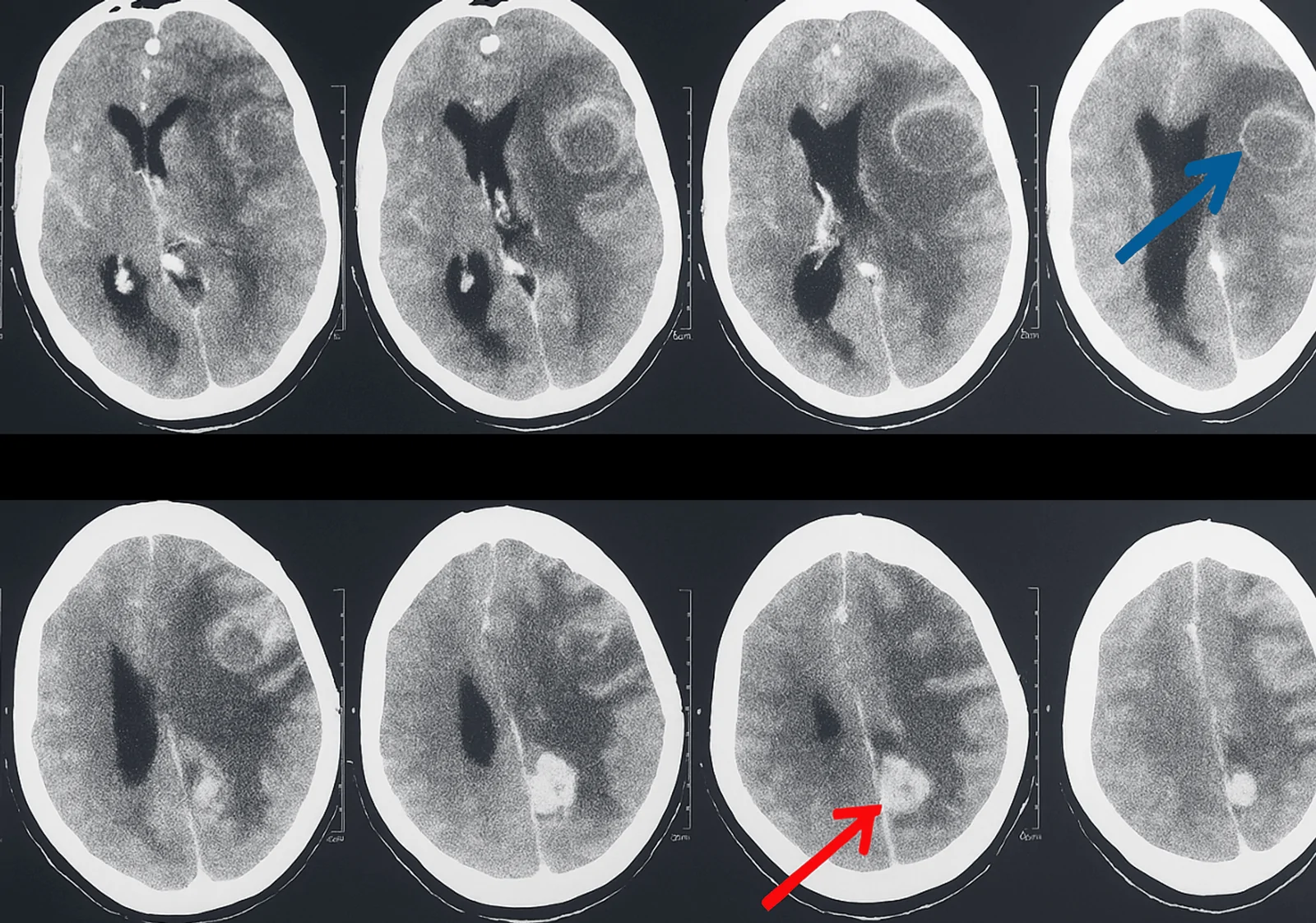
Contrast-enhanced brain CT revealing dual lesions with distinct radiologic characteristics. Contrast-enhanced axial brain CT showing two lesions: Blue arrow: well-circumscribed, round hypodense lesion in the left frontoparietal region with a ring-like hyperdense margin and surrounding “finger-like” perilesional edema, causing a mass effect on the midline and ipsilateral ventricle; Red arrow: irregular, poorly defined hyperdense lesion adjacent to the superior sagittal sinus, possibly adherent to it, also associated with glove-finger–shaped perilesional edema.

**Figure 2:**
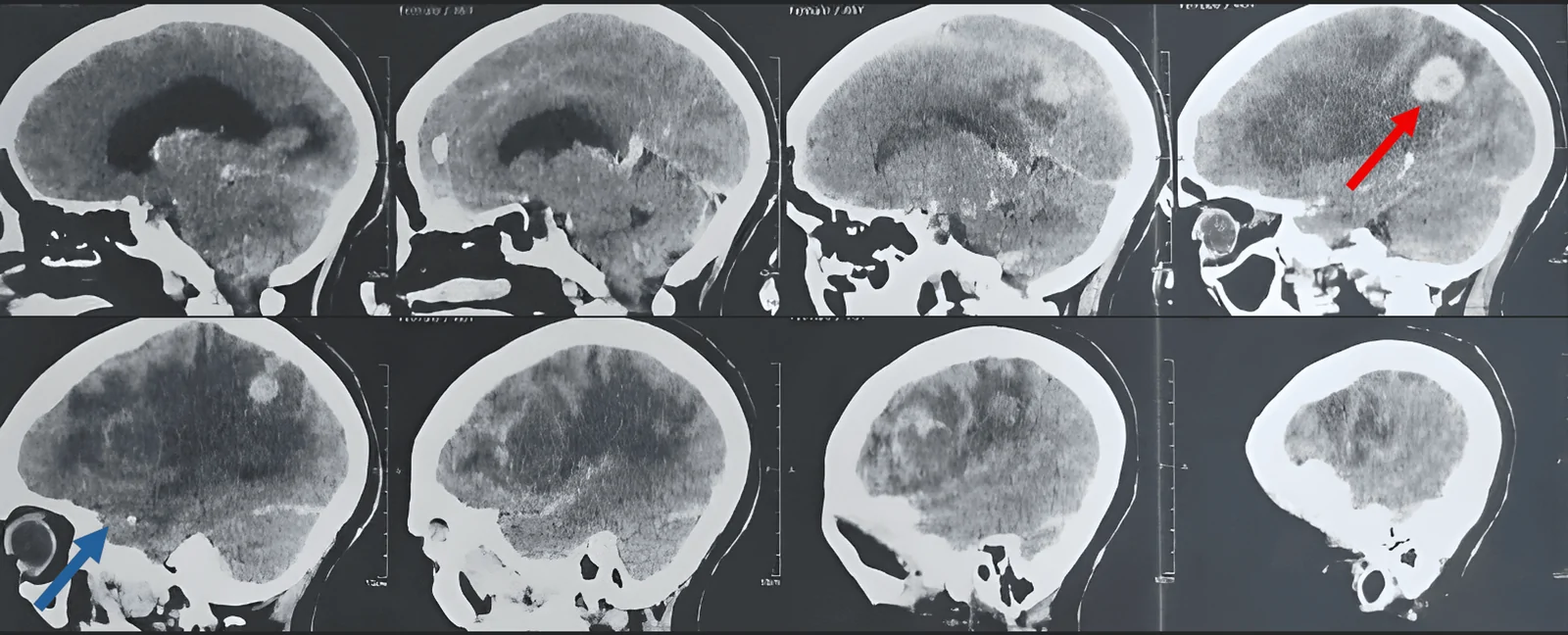
Contrast-enhanced sagittal brain CT demonstrating dual lesions with distinct morphologic features. Contrast-enhanced sagittal brain CT showing the same lesions as in Figure 1: Blue arrow: well-circumscribed, round hypodense lesion in the left frontoparietal region with a ring-like hyperdense margin and surrounding “finger-like” perilesional edema, causing a mass effect on the midline and ipsilateral ventricle; Red arrow: irregular, poorly defined hyperdense lesion, also associated with perilesional edema.

**Figure 3:**
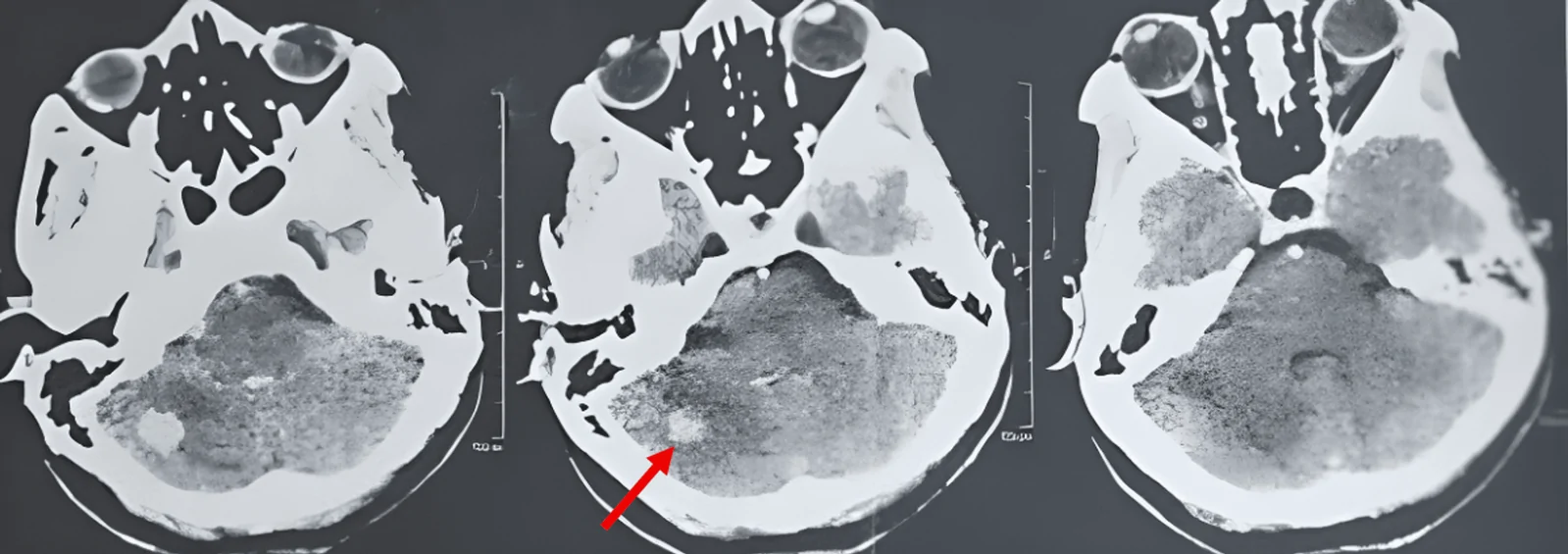
Contrast-enhanced brain CT revealing a right cerebellar lesion with irregular margins and perilesional edema. Contrast-enhanced axial brain CT showing a hyperdense, poorly defined lesion with irregular margins in the right cerebellar hemisphere, associated with mild perilesional edema.

No further workup was performed, as the patient was admitted in an emergency setting. The patient underwent emergency surgery, targeting the largest lesion situated in the left frontoparietal region. A puncture and aspiration were performed; the lesion was found to be a purulent collection, macroscopically yellow-green. The material was sent to the laboratory for cytobacteriological analysis.

Pathological examination revealed purulent material, yet microbiological culture remained sterile, suggesting either prior antibiotic therapy or the presence of pathogens difficult to culture. At the time of admission, no antibiotics were administered, although prior oral antibiotic therapy for a different infection remains a possibility considering the circumstances.

The patient was initiated on triple antibiotic therapy (metronidazole, third-generation cephalosporin, and gentamicin), combined with corticosteroid therapy and supportive intensive care measures. Despite treatment, she showed no clinical improvement and remained intubated, ventilated, and sedated, with bilaterally dilated, non-reactive pupils. Given the patient’s critical neurological condition and rapid postoperative deterioration, no postoperative brain CT scan was performed.

Despite maximal medical and supportive management, the patient failed to improve and subsequently passed away. In the absence of postoperative imaging, the exact cause of the patient's deterioration and death could not be determined, and a postoperative complication, such as surgical-site hemorrhage, could not be ruled out. This represents an important limitation of the present case report.

## Discussion

Cerebral abscesses are rare but serious intracranial infections that can present with clinical and radiological features indistinguishable from those of brain tumors [3], posing significant diagnostic challenges. The differential diagnosis between cerebral abscesses and brain tumors is crucial, as the management strategies for these conditions differ markedly [4].

Patients with cerebral abscesses often present with nonspecific symptoms, such as headache, fever, nausea, vomiting, and focal neurological deficits [1]. In our case, the patient exhibited an incomplete syndrome of intracranial hypertension, including headaches and vomiting for three weeks, which progressed to dysarthria and right-sided hemiplegia. This progression underscores the importance of considering infectious etiologies in patients presenting with neurological deterioration.

The most common pathogens responsible for cerebral abscesses include *Streptococcus* spp., *Staphylococcus* spp., and Gram-negative enteric bacteria [5,6]. A meta-analysis by Bodilsen et al. reported that *Streptococcus* spp. was identified in 32% of cases, *Staphylococcus* spp. in 14%, and Gram-negative enteric bacteria in 9% of cases [7]. These organisms can reach the brain via contiguous spread from adjacent infections, hematogenous dissemination, or direct inoculation during neurosurgical procedures [8].

Radiologically, cerebral abscesses often present as ring-enhancing lesions with surrounding edema on contrast-enhanced imaging studies. In this case, MRI could not be performed, limiting further characterization of the lesions and refinement of the differential diagnosis. Advanced imaging techniques, such as diffusion-weighted imaging (DWI), magnetic resonance spectroscopy (MRS), and perfusion-weighted imaging (PWI), can aid in distinguishing cerebral abscesses from other intracranial lesions, including necrotic tumors. For instance, cerebral abscesses typically demonstrate restricted diffusion on DWI, a feature less commonly seen in necrotic tumors [3].

The management of cerebral abscesses involves a combination of surgical intervention and antimicrobial therapy: (i) Surgical options include aspiration, drainage, or excision, depending on the size, location, and accessibility of the lesion [9]. (ii) Empirical antibiotic therapy should be initiated promptly, tailored based on the most likely pathogens and adjusted according to culture results [10].

Standard empiric therapy generally consists of a third-generation cephalosporin combined with metronidazole to cover streptococci, enterobacteria, and anaerobes. In cases of post-traumatic or post-neurosurgical abscesses, vancomycin may be added to cover *Staphylococcus aureus*, including methicillin-resistant *Staphylococcus aureus* (MRSA) strains. The duration of therapy typically extends for six to eight weeks, with intravenous treatment initially followed by an oral course guided by clinical and radiological response [9]. Adjunctive corticosteroids may be considered to reduce cerebral edema and mass effect [11].

Despite advances in imaging and surgical techniques, the prognosis for patients with cerebral abscesses remains guarded. A systematic review by Choucha et al. highlighted that, despite appropriate treatment, outcomes can be poor, particularly in patients with delayed diagnosis or those presenting with significant neurological deficits [3]. Early recognition and intervention are paramount to improving survival and functional outcomes. The inability to obtain an MRI in this patient underscores a limitation in diagnostic precision that may have affected management decisions.

The coexistence of a brain abscess with cerebral metastases is uncommon. In this case, there was no recent infection and no identifiable entry point, such as an otorhinolaryngological or dental focus. The development of an abscess in this context may be explained by tumor-related necrosis creating a local environment favorable to bacterial colonization, by hematogenous seeding from subclinical or remote infections, or by local disruption of the blood-brain barrier induced by the metastatic lesion. Additionally, immunosuppression related to malignancy could increase susceptibility to opportunistic infections, even in the absence of overt infectious symptoms. 

Similar to previously reported cases, such as the one by Martínez and Piaggio [12], the coexistence of a brain abscess within a metastatic lesion is a rare occurrence. In both cases, the abscess developed in the absence of a clearly identifiable source of infection, emphasizing the role of factors such as tumor necrosis, local disruption of the blood-brain barrier, and potential hematogenous seeding from subclinical infections. While advanced imaging techniques, including diffusion-weighted MRI, were highlighted by Martínez and Piaggio as useful for distinguishing abscesses from necrotic metastases, our case further underscores the importance of considering superimposed infection when evaluating rapidly evolving lesions, even in patients without overt infectious symptoms.

This case underscores the necessity of considering infectious etiologies in the differential diagnosis of intracranial mass lesions and the importance of rapid multidisciplinary management. Early recognition and treatment remain key to improving survival and functional outcomes in patients with cerebral abscesses.

## Conclusions

Cerebral abscesses can mimic brain tumors both clinically and radiologically, presenting a significant diagnostic challenge. Prompt recognition, surgical intervention when indicated, and appropriate antimicrobial therapy are essential components of management. This case highlights the importance of considering infectious etiologies in patients presenting with intracranial lesions, even in the absence of fever or an identifiable infectious source, and underscores the value of timely multidisciplinary assessment in guiding diagnosis and treatment.
